# Supplementary material for: Outcomes of Universal Newborn Screening Programs: Systematic Review
Source: J Clin Med. 2021 Jun 24;10(13):2784. doi: 10.3390/jcm10132784 (PMC8268039; doi:10.3390/jcm10132784)
Supplement: Supplementary file 1 [file jcm-10-02784-s001.zip › jcm-1241700-supplementary.pdf]

Supplementary Materials

Supplementary Table S1. Age of Identification by type of screen.

Study	Location	Study Design	Study Setting	Study Population	Outcomes in Intervention Group (UNHS)			Age of ID	Outcomes in Comparison Group (No UNHS)			Age of ID	Absolute/ Relative Effect (95% CI)
					**Number of Participants**	**No. of Participants with Outcome (PBHL)***	**% with Outcome**	**UNHS**	**Number of Participants**	**No. of Participants with Outcome (PBHL)**	**% with Outcome**	**No UNHS**	
Kennedy et al. (2006) [1]	UK	Cohort study: UNHS vs No UNHS (No UNNHS=DS Nested case control?	UNHS(INP) and diagnosis OUTP	Original cohort consisted of 157,000 newborns born between 1992–1997	68,714 infants born during periods in which UNHS was in place	49/77 identified	64% 49/77) of ID HL	No mean/median AgeID 67% (41/67) ID < 9 months	88,019 infants born during periods without UNHS n = 168 Identified	59/91 Identified	51–57% (46–52/91 ID HL)	No mean/ median AgeID 27% (16/59) ID < 9 months	Absolute difference: 40% Mean difference/adjusted mean difference (95% CI)
Uus & Bamford (2006) [2]	UK	Cohort	Screen: INP AudDx, OUTP	169,487 infants screened	169,487 infants screened	n = 169 PCHL		Median = 10 weeks (2.5 months)					
Wood, Sutton, and Davis (2015) [3]	UK	Population Birth cohorts 06/07 annually to 13/14	Screen: INP AudDx, AudAMP, Rx: OUTP	2006–2013 UK stats 660,000 x 8 years	5,280,000 births	5,280,000	100% IDeded n = 4737 2007–14		2013–14 Median age ID: 49 days (1.6 months)				
Yoshinaga-Itano (2000, 2001) [4,5]	USA Colorado	Case control population	UNHS INP Diagnosis: OUTP	Approximately 148,000 births screened (Mehl & Thomson 2002)[6]	Approximately 148000 screened	UNHS: n = 25 No UNHS n = 25 Matched groups 54/89 IDed = 61%	4/54 ID >6 months	Median: 5 weeks (1.25 months) 93% AgeID< 6 months 7% AgeID>6 months	Approximately 152,000 births not screened	52 identified Unknown—total ID of unscreened	4/ 52 ID < 6 months 48/52 ID > 6 months	Median: 23 months 8% IDed < 6 months 92% IDed > 6 months	Wilcoxon signed rank test (Z = 4.24, p<0.001) 95% CI
Wake et al. (2016) [7]	Australia	Quasi-randomized	Screen INP Diagnosis: OUTP	296,378 infants screened	172,253 screened	69/179 (eligible)	39% of eligible	8.1 months (mean) age of ID	123,855 No screen	RFS: 65/134 No info for No Screen—total # identified 1991–1993	49% of eligible	No UNHS Screen: RFS-16.2 months (mean) No Screen: 22.5 months (mean)	UNHS-RFS: −6.4 (−11.0 to −1.8) UNHS—No Screen: −14.4 (−19.3 to −9.6) <0.001

Age of ID: Age of identification, AudAMP: Amplification, Rx: Intervention, AudDx: Audiological diagnosis, CI: Confidence interval, DS: Distraction Screening HL: Hearing loss, INP: inpatient, OR: Odds Ratio, OUTP: outpatient, PBHL: Permanent bilateral hearing loss, RFS: Risk Factor Screen, UK: United Kingdom, UNHS: Universal newborn hearing screening, USA: United States of America.

Supplementary Table S2. Proportion by age of identification at 12, 9, 6, 3 months.

Study	Location	Study Design	Study Setting	Study Population	Outcomes in Intervention Group—UNHS				Outcomes in Comparison Group—No UNHS				Absolute/ Relative Effect (95% CI)
					Number of Participants	No. of Participants with Outcome (PBHL)*	% with Outcome	Proportion UNHS	Number of Participants	No. of Participants with Outcome (PBHL)	% with Outcome	Proportion No UNHS	
Kennedy et al. (2006) [1]	UK	Cohort study: UNHS vs no UNHS (no UNHS=s DS	UNHS (INP) and diagnosis (OUTP)	Original cohort consisted of 157,000 newborns born between 1992–1997	68,714 infants born during periods in which UNHS was in place	49/77 identified	64%	<9 months: 67%<br>>9 months: 33%	88,019 infants born during periods without UNHS<br>n = 168 IDed	46/91 IDed	51–57%	<9 months: 27%<br>>9 months: 73%	Absolute difference: 40%<br>Mean difference/adjusted mean diff (95% CI)
Yoshinaga-Itano (2000, 2001) [4,5]	USA Colorado	Case control population	UNHS inpatient Diagnosis: outpatient	Approximately 148,000 births screened (Mehl & Thomson 2002) [6]	Approximately 148,000 screened	UNHS: n = 54<br>No UNHS n = 52<br>54/89 IDed = 61%	50 of 54 AgeID < 6 months<br>4/54 AgeID >6months	<6 months: 93%<br>>6 months: 7%	Approximately 152,000 births not screened	52 identified Unknown—total IDed of NS	4/ 52 >6 months<br>48/52 >6 months	AgeID<6 months: 8%<br>AgeID>6 months: 92%	Wilcoxon signed rank test<br>(Z = 4.24, p < 0.001)<br>95% CI
Weichbold et al. (2006) [8]	Austria	1990–2003 Retrospective case-control: ENT clinic database	1250,000 births total<br>25% Approximately 312,220 births	321 identified HL of<br>Of 1,250 identified	n = 167	About 156,000	100%	<3 months: 35%<br>>3 months: 65%<br><6 months: 69%<br>>6 months: 31%<br><12 months: 81%<br>>12 months: 19%	n = 154	About 156,000	100%	<3 months: 2%<br>>3 months: 98%<br><6 months: 6%<br>>6 months: 94%<br><12 months: 12%<br>>12 months: 88% n = 167	No statistical analysis

CI: Confidence interval, ID: identification, IDed: Identified; INP: In Patient; NS=No Screen; OR: Odds Ratio, OUTP: Out Patient; PBHL: Permanent bilateral hearing loss, RFS: Risk Factor Screen, UK: United Kingdom, UNHS: Universal newborn hearing screening, USA: United States of America.

Supplementary Table S3. Early identification, age of amplification and age at early intervention outcomes of UNHS.

Study	Location	Study Details					Outcomes		
		Study Design	Setting	Population	Participants (EG = Experimental Group, CG = Control Group)	Screen Method	Age ID	Age Amp	Age Rx
Kennedy et al. (2006) [1]	UK	Cohort	Screen: Inpatient AudDx, Aud Amp, RX: OUTP	1993–6<br>157,000 infants screened<br>(87% coverage of births)<br>1.5% false positive	EG: *n* = 120 PCHL<br>(*n* = 63 NH;<br>*n* = 57 UNHS)<br>CG: *n*=60 NI<br>ID < 9 months (*n* = 57)<br>ID > 9 months (*n* = 63)	Two-stage TEOAE-AABR	Median = 10 months (PCHL group)<br>Information about the <9-month versus >9-month groups	Median = 15 months (PCHL group)	Median = 13 months (PCHL group)<br>87% by 3 months after ID<br>Enrollment management median 15 months
Uus & Bamford (2005) [2]	UK	Cohort	Screen: INP AudDx, AudAmp, Rx: OUTP	169,487<br>infants screened	*n* = 169 PCHL	WB = OAE, if fail AABR<br>NICU = TEOAE+AABR	Median = 10 weeks (2.5 months)	Median = 16 weeks (4 months)	Median = 10 weeks (2.5 months)
Wood Sutton & Davis (2015) [3]	UK	Population Birth cohorts 06/07 annually to 13/14	Screen: INP AudDx, AudAMP, Rx: OUTP	2006–2013 UK stats<br>660,000 x 8 years	5,280,000 births	WB = OAE, if fail AABR<br>NICU = TEOAE + AABR	2013–14 Median age of ID: 49 days (1.6 months)	2013–14 Median days HA fit: 82 days<br>(2.7 months)	2013–14 Median days referral EI: 50 days<br>(1.7 months)
Wake et al. (2016) [7]	Australia	Quasi-randomized	Screen: INP AudDx, AudAMP, Rx: OUTP	2003–2005<br>172,523 babies NSW<br>123,855 Victoria	EG: UNHS *n* = 49, RFS *n* = 56<br>Control: No UNHS *n* = 57 (1991–3)	No info on technology used for screen	UNHS 8.1 months<br>RFS: 16.2 months<br>No UNHS: 22.5	UNHS:13.5 months<br>RFS: 17.9 months<br>No UNHS: 24 months	No info EI management
Sininger et al. (2009) [9]	USA	Cohort	Screen: OUTP AudDx,<br>AudAMP, RX: OUTP	No info on births screened	EG: UNHS *n* = 47 (*n* = 39 fail, *n* = 8 pass)<br>Control: No UNHS *n* = 17	No info on technology used for screen	UNHS= 3 months (median)<br>Fail=2.4 months<br>Pass=22.5 months<br>No UNHS=27.8	UNHS= 5.6 (median)<br>Fail=4.9 months<br>Pass=24.9 months<br>No UNHS *n*=29.1	UNHS 10.58 months (median) Fail=8.9 months<br>Pass=40.46 months<br>No UNHS=30.5 months
Dalzell et al. (2000) [10]	USA	Cohort	Screen: INP AudDx, AudAMP, RX: OUTP	1995–6 43,311 newborns	EG: *n* = 85 PCHL<br>(NICU = 61%<br>At risk = 67%<br>WBN = 39%	TEOAE &/or AABR	Median age of ID 3 months<br>UHL 3.5 months<br>BHL 3.0 months<br>80% children w/HL ID by 5 months<br>Age of ID: WBN < NICU, severe/profound < mild moderate<br>No significant difference risk vs. No Risk	Median age of HA fit 7.5 months	Median age RX enrollment: 3 months

Yoshinaga-Itano et al. (2001) [5]	USA	Cohort	AudDx, Aud AMP, RX: OUTP	# Screened births not provided	EG: UNHS: n = 25<br>PRUNHS:<br>n = 29<br>Control: No UNHS: n = 25<br>PRNS n = 77<br>Born before 1992<br>n = 118	No info on technology used for screen Technology in Colorado reported by Mehl & Thomson [6] was AABR, TE-OAE, AABR+TE-OAE	UNHS: Age of ID<6 months 84%<br>Median 5 weeks<br>NS: Age of ID< 6 months 8%<br>NS Median<br>23 months	Age AMP—not reported<br>Yoshinaga-Itano et al 1998 [11] reported AgeAMP within 2 months of age of ID for EID children and 1 month of age of ID for LID children	EI 87% within 2 months Age of ID UNHS by 13 weeks NS 25 months

AABR: Automated auditory brain stem, AMP: Amplification, AUD: audiology, BHL: Bilateral hearing loss, CG: control group, CQ: Cognitive Quotient, DS: Distraction Screen, EG: Experimental group, EI: Early Intervention, EID: Early Identified, HA: Hearing Aid Fit, ID: Identification, INP: In Patient; LID: Late-identified, MLE: Mother's level of education, NICU: newborn intensive care unit, NS: No Screen, OUTP: Out Patient; PCHL: Permanent Childhood Hearing Loss, PRNUNHS: Parent Report No UNHS, PRUNHS: Parent Report UNHS, RFS: Risk Factor Screen, RX: Intervention, TEOAE: Transient evoked otoacoustic emissions, UHL: Unilateral hearing loss, UK: United Kingdom, UNHS: Universal newborn hearing screening, USA: United States of America, 'WB-OAE: well baby nursery-otoacoustic emissions.

Supplementary Table S4. Language/Reading Outcomes by UNHS vs No UNHS.

Study	Location	Study Design	Study Setting	Study Population	Outcomes in Intervention Group—UNHS			Lang: REC EXP	Outcomes in Comparison Group—No UNHS			Lang: REC EXP	Absolute/ Relative Effect (95% CI)
					# Participants	# Participants w/outcome (PBHL)	% with Outcome	UNHS	# Participants	# Participants w/outcome (PBHL)	% with Outcome	No UNHS	
Kennedy et al. (2006) [1]	UK	Cohort study: UNHS vs no UNHS	UNHS: INP DevAss: OUTP)	Original cohort: 157,000 newborns born 1992–1997	*n* = 57	TROG [12]: *n* = 46 BPVS [13]: *n* = 52	57/77 74%	Rec Lang: TROG [12]: -1.71 (1.78) BPVS [13]: Aggregate −1.89 (1.65) Aggregate -NV −0.94 (1.45)	*n* = 63 *n* = 60 NH	TROG [12]: *n*=52 BPVS [13]: *n* = 52	TROG [12] 57% BPVT [13] 57%	Rec Lang-TROG [12] −2.1(1.75) Rec Lang-BPVS [13]: Aggregate −2.32 (1.61) Aggregate - NV −1.67 (1.29)	TROG [12]:Adjusted mean diff 0.59 (=.01-1.19) *p*=0.05 BPVS [13]: Aggregate 0.56 (0.03 to 1.08) *p* = 0.04 Aggregate -NV: 0.60 (0.07 to 1.13) *p* =0.03
McCann et al. (2009) [14]	UK Wessex Yield: 120/168 (71.4%)	Cohort UNHS vs. No UNHS CA: Mean age at testing 7.9 Range 5.4 to 11.7 years	Screen: INP Dev Assessment: OUTP	1993–6 157,000 infants screened (87% coverage of births) 1.5% false positive Two-stage TEOAE-AABR	EG: *n* = 120 UNHS *n* = 57 No UNHS *n* = 63	VABS *n* = 50	*n* = 50—57	VABS [15] Group mean z score: −0.21 (1.13) WORD [16] reading Aggregate: −0.42 (−1.30)	*n* = 63	VABS [15] *n* = 48	76%	VABSCOMM [15]: −0.64 (1.39) WORD [16] reading Aggregate: −0.74 (1.16)	VABSCOMM [15]: 0.51 (0.06 to 0.95) *p*=0.026 WORD [16] reading Aggregate: 0.52 (−0.21 to 1.24,) *p*= 0.16
Pimperton et al (2016) [17]	UK Wessex cohort	Cohort study: UNHS vs No UNHS Age of ID<9 months vs Age of ID >9 months	UNHS: INP DevAss: OUTP	Original cohort consisted of 157,000 newborns born between 1992–1997	68,714 infants born during periods in which UNHS was in place								
				Age of ID	YARC [18] RDGCOMP YARC [18] RDGSUM	*n* = 28	36%	YARC [18] RDGCOMP: −0.63 (1.63) YARC [18] RDGSUM −0.56 (1.37)	88,019 infants *n* = 91 infants with HL born with No UNHS	*n* = 37	41%	YARC [18] RDGCOMP: −1.74 (1.50) YARC [18] RGSUM: −1.36 (1.44)	YARC [18]RDGCOMP 1.17 (0.36 to 1.97) *p* =0.005 YARC [18] RDGSUM 0.96 (0.24 to 1.68) *p* = 0.01
				UNHS	YARC [18] RDGCOMP YARC [18] RDGSUM	*n* = 33 (RDGCOMP/ RDGSUM) *n* = 31 (RDGACC)	*n* = 33/77 45%	RDGCOMP −1.15 (1.87) RDGSUM −0.97 (1.51) RDGACC−1.43 (1.59)	*n* = 91	*n* = 28	*n* = 32/91 (RDGCOMP & RDGSUM) *n* = 29/91 (Accuracy)	RDGCOMP −1.37 (1.39) RDGSUM: −1.07 (1.43) RDGACC: −1.47 (1.58)	Not significant: RDGCOMP: 0.15 (−0.75 to 1.06) *p* = 0.73 RDGSUM: 0.22 (−0.58 to 1.03) *p* = 0.58 RDGACC: 0.09 (−0.76 to 0.93) *p* = 0.84

Yoshinaga-Itano (2000, 2001) [4, 5]	USA<br>Colorado	Case control<br>Population<br>Age at testing<br>12–60 months	UNHS<br>INP<br>Dev Ass:<br>OUTP	Approximately 148,000 births screened (Mehl & Thomson 2002) [6]	Approximately 148000 screened	UNHS: n = 25<br>28%	25/89	Screen:<br>CDI [19] ELQ: 82.9<br>CDI [19] Rec Lang LCQ :81.5<br>Total Lang:<br>82.2	No UNHS<br>n = 25	No info total# not screened for HL	Info not provided	ELQ:<br>62.1<br>LCQ Rec Lang:<br>66.8<br>Total Lang: 64.4	Wilcoxin signed rank test<br>(Z = 4.24, p < 0.001)<br>95% CI
						UNHS<br>n = 54	54/89	Screen:<br>81.0%–82.4%	Screen&Probable Screen<br>n = 54<br>61%	No Screen&Probable No Screen<br>n = 77	Info not provided	Probable No Screen (same birth cohort as Screen):<br>Total Lang: 62.1<br>No Screen <1992 before UNHS:<br>Total Lang: 55.7	Screen/No Screened: n=131<br>Likelihood of NML range LQ>=80<br>OR: 2.54<br>Lower 1.6<br>Upper: 4.2<br>Screen/No Screened (include born <1992): n=249<br>Likelihood of NML range LQ>=80<br>OR:2.1<br>Lower: 1.53<br>Upper: 2.88
Wake et al (2016) [7]	Australia	Quasi-randomized<br>Age at testing<br>3–5 years	Screen<br>INP<br>Diagnosis:<br>OUTP	296,378 infants screened	172,253 screened	69/179	39% of invited	PLS [20] Rec Lang:<br>81.8 (No screen)<br>83.0 (RFS)<br>88.9 (UNHS)<br>PLS [20] Exp Lang:<br>74.9 (NS)<br>80.7 (RFS)<br>89.3 (UNHS)<br>PPVT [21] Rec Voc:<br>79.4 (NS)<br>80.7 (RFS)<br>91.5 (UNHS)	123,855 No screen	RFS: 65/134<br>No info for–total # IDed 1991–1993	49% of invited	No UNHS<br>RFS:<br>Exp Lang: 80.0<br>Rec Voc:<br>82.9<br>No UNHS:<br>OPP before UNHS<br>Rec Lang: 81.8<br>Exp Lang: 74.9<br>Rec Voc:79.4	UNHS-RFS:<br>Exp Lang: −8.2 (0.5 to 15.9) p =0.04<br>Rec Voc:<br>8.1 (0.8 to 15.4) p =0.03<br>UNHS vs. OPP<br>Rec Lang:<br>7.0 (0.2–13.8) p=0.05<br>Exp Lang:<br>14.4 (7.3–21.5) p <0.001<br>Rec Voc:<br>12.1 (5.9–18.4 p <0.001

BVPS: British Vocabulary Picture Scale, CDI: Child Development Inventories; DevAss: Developmental Assessment; ELQ: Expressive Language Quotient, Exp Lang: Expressive Language; INP: Inpatient, LCQ: Language Comprehension Quotient, NS: No Screen, OPP: Opportunistic identification; OUTP: Outpatient, PBHL: Permanent bilateral hearing loss, PLS: Preschool Language Scale, PPVT: Peabody Picture Vocabulary Test, Rec Lang: Receptive Language; Rec Voc: Receptive Vocabulary; RDGACC: Reading Accuracy; RDGCOMP: Reading Comprehension; RDGSUM: Reading Summarization; RFS: Risk Factor Screen: TROG: Test for Reception of Grammar; UK: United Kingdom, UNHS Universal newborn hearing screening, USA: United States of America, VABSCOMM: Vineland Adaptive Behavior Scales Communication, YARC: York Assessment of Reading for Comprehension. .

Supplementary Table S5. Language/Reading Outcomes by Age of Identification.

Study	Location	Study Design	Study Setting	Study Population	Outcomes in Intervention Group—UNHS				Outcomes in Comparison Group—No UNHS				Absolute/ Relative Effect (95% CI)
					Number of Participants	No. of Participants with Outcome (PBHL)*	% with Outcome	Lang/ Rdg	Number of Participants	No. of Participants with Outcome (PBHL)	% with Outcome	Lang/ Rdg	
Kennedy et al (2006) [1]	UK	Cohort study: UNHS vs No UNHS	UNHS: INP<br>DevAss: OUTP	Original cohort consisted 157,000 newborns born between 1992–1997	68,714 infants born w/UNHS	41–49/77	41–49/77<br>53–64%		88,019 infants born with No UNHS	AgeID >9months<br>*n* = 46%	46–52/91<br>51–57%		Mean difference/adjusted mean diff (95% CI)
				AGEID < 9 months<br>AgeID > 9 months	69,714 infants born w/UNHS	Rec Lang<br>TROG [12]<br>n=44<br>BPVS [13] n=45<br>Aggregate Score<br>Aggregate Score minus NV	AgeID <9 months 45/77<br>58%	Rec Lang:<br>TROG [12]:<br>−1.46 (1.50)<br>BPVS [13]:<br>−1.86 (1.40)<br>Aggregate Score:<br>−1.76 (1.47)<br>Aggregate—NV:<br>−0.82 (1.23)	88,019 infants born with No UNHS	54/91 TROG<br>AgeID >9 months<br>59%<br>55/91 BPVS<br>60%	59–60%	Rec Lang:<br>TROG [12]:<br>−2.25 (1.91)<br>BPVS [13]:<br>−2.36 (1.65)<br>Aggregate Score:<br>−2.38 (1.72)<br>Aggregate—NV:<br>−1.68 (1.44)	Rec Lang:<br>TROG [12]:<br>0.90 (0.32–1.47)<br>*p*<0.003<br>BPVS [13]:<br>0.64 (0.13–1.16) *p*< 0.02<br>Aggregate Score:<br>0.76 (0.26–1.27) *p*< 0.004<br>Aggregate -NV:<br>0.82 (0.31–1.33)<br>*p* < 0.002
				AgeID < 9 months<br>AgeID > 9 months	69,714 infants born w/UNHS	Exp Lang<br>RBST [22]: 5 long Sent<br>Aggregate score<br>Aggregate minus NV	RBST AgeID <9 months<br>39/77<br>51%	Exp Lang<br>RBST [22]:<br>−0.73 (1.32)<br>Aggregate score:<br>−0.59 (1.31)<br>Aggregate—NV:<br>0.14 (1.29)	88,019 infants born with No UNHS	RBST 48/91<br>AgeID > 9months<br>53%	53%	Exp Lang<br>RBST [22]:<br>−1.23 (1.15)<br>Aggregate score:<br>−1.07 (1.21)<br>Aggregate—NV:<br>−0.50 (1.34)	RBST [22]<br>0.54 (0.05–1.04)<br>*p* < 0.03<br>0.47 (−0.14–1.08)<br>*p* < 0.13<br>0.50 (<0.01–1.01)<br>*p* < 0.05<br>0.70 (0.13–1.26)<br>*p* < 0.02
McCann et al., (2009) [14]	UK	Cohort study: UNHS vs No UNHS	UNHS: INP<br>DevAss: OUTP	Original cohort consisted of 157,000 newborns born between 1992–1997	168,714 infants born w/UNHS	Age of ID <9 months:<br>43-45/77	Age of ID <9 months:\43-45/77<br>56–58%	WORD[16] basic reading:<br>WORD [16]<br>RDGCOMP:<br>Aggregate:<br>VABS 57]:	88,019 infants born with No UNHS	AgeID >9months<br>*n* = 54%	Age of ID > 9 months:<br>54–57/91<br>59–63%	WORD [16] basic reading:<br>WORD [16]<br>RDGCOMP<P:<br>Aggregate:<br>VABS [13]:	Mean difference/adjustment mean (95% CI)<br><br>Aggregate RBST [22] reading and VABS [15] communication

				Age of ID	WORD[16] reading ability<br>Basic reading<br>RDGCOMP<br>Aggregate reading<br>VABS [15] comm	$n = 43–45$	AgeID < 9 months: 43–45/77<br>56–58%	WORD [16] basic reading:<br>−0.54 (1.18)<br>WORD [16] RDGCOMP:<br>−0.71 (1.18)<br>Aggregate:<br>−0.65 (1.14)<br>VABSCOMM [15]:<br>−0.13 (1.16)	88,019 infants born w/o UNHS	52-65%	Age of ID >9months:<br>51-57/91<br>59–63%	WORD[16] basic reading:<br>−1.02 (1.24)<br>WORD [16] RDGCOMP:<br>−1.09 (1.16)<br>Aggregate:<br>−1.10 (1.21)<br>VABSCOMM [15]:<br>= 0.65 (1.33)	WORD [16] basic reading:<br>0.55 (0.17–0.93)<br>$p < 0.005$<br>WORD [16] RDGCOMP:<br>0.48 (0.09–0.86)<br>$p < .016$<br>Aggregate:<br>0.51 (0.15–0.87)<br>$p<.006$<br>VABSCOMM [15]:<br>0.56 (0.12–1.00)<br>$p<0.013$
Worsfold et al., (2010) [23]	UK	Cohort study: UNHS vs No UNHS	UNHS: INP<br>DevAss: OUPT	Original cohort consisted 157,000 newborns born between 1992–1997	68,714 infants born w/UNHS	AgeID < 9 months<br>41/77	AgeID < 9 months :<br>41/77<br>53%		88,019 infants born w/<br>No UNHS		Age of ID; > 9 months:<br>n=48/91<br>45%		Logistic regression<br>ID</>9 months/ID>9 months
				Age ID<br># sent<br># categories of morphological endings (MORPHEND) high frequency (HF)<br>Narrative structure (NARRSTRUC)<br>Narrative content score (NARRCON)	68,714 infants born w/UNHS	AgeID < 9 months<br>$n = 41$	AgeID < 9 months<br>53%	#sent:<br>14.56 (6.07)<br>#Morphend HF:<br>23.34 (11.74)<br>NARRSTRUCt score:<br>6 (14.6)<br>NARRCON score:<br>10 (24.4)	88,019 infants born w/ No UNHS	48/91<br>AgeID > 9months<br>53%		#sent:<br>13.06 (4.86)<br>#MORPHEND HF:<br>19.02 (10.63)<br>NARRSTRUC score:<br>22 (45.8)<br>NARRCON score:<br>19 (39.6)	#sent:<br>2.86 (0.49–5.24) $p<$ 0.019<br>#MORPHEND HF:<br>6.64 (1.96–11.31)<br>$p< 0.006$<br>NARRSTRUC score:<br>3.03 (1.09–8.46)<br>$p < 0.034$<br>NARRCON score:<br>4.43 (1.52–12.89)<br>$p < 0.006$
Pimperton et al (2016) [17]	UK	Cohort study: UNHS vs no UNHS	UNHS:<br>INP<br>DevAss: OUTP	Original cohort consisted of 157,000 newborns born between 1992–1997	68,714 infants born during periods in which UNHS was in place	Age ID < 9 months:<br>27-28/77<br>35–36%	Age of ID < 9 months:<br>35–36%		88,019 infants/91 with HL born with No UNHS	AgeID > 9months<br>33–37/91<br>36–41%	27-28/91<br>30–31%		Mean difference/adjusted mean diff (CI 95%)
				Age of ID	YARC [18]<br>RDGCOMP	$n = 28$	36%	YARC [18]<br>RDG COM{:<br>−0.63 (1.63)	88,019 infants	$n = 37$	41%	YARC [18]<br>RDGCOMP:<br>−1.74 (1.50)	YARC<br>RDGCOMP [18]

					YARC [18] RDG SU<			YARC [18] RDGSUM: −0.56 (1.37)	n = 91 infants w/HL born w/ No UNHS			YARC [18] RDGSUM: −1.36 (1.44)	1.17 (0.36–1.97) p = .005 YARC RDGSUM [18] 0.96 (0.24–1.68) p = 0.01
Yoshinaga-Itano (2020) [24]	USA Colorado	Case control population	UNHS :INP DevAss: OUTP	Approximately 148,000 births screened (Mehl & Thomson 2000) [6]	Child Development Inventory [19]	n = 72 AgeID < 6 months	No info provided	CDI [19] Rec Lang: 92.2 CDI [19] Exp Lang: 90.5 Total Lang: 91.3	No info provided	n = 78 AgeID > 6 months	No info provided	CDI [19] Rec Lang: 71.7 CDI [19] Exp Lang: 68.7 Total Lang: 70.2	CDI [19] Rec Lang: $F[1, 82]$ 24.5, $p < 0.001$ CDI [19] Exp Lang: $F[1, 82]$ 25.8. $p < 0.001$ Total Lang: $F[1, 82]$ 29.6, $p < 0.001$

Age ID: Age of Identification; BVPS: British Vocabulary Picture Scale, CDI: Child Development Inventories, DevAss: Developmental Assessment; HL=hearing loss; HF=high frequency; INP: Inpatient, , MORPHEND: Morphological Endings; NARRCON: Narrative Content; NARRSTRUC: Narrative Structure; NV: Nonverbal, OUTP: Outpatient, PBHL: Permanent bilateral hearing loss, RDGCOMP: Reading Comprehension; RDGSUM: Reading Summarization; RBST: Renfro Bus Story Test, TROG: Test for Reception of Grammar, UK: United Kingedom, UNHS: Universal newborn hearing screening, USA: United States of America, VABSCOMM: Vineland Adaptive Behavior Scales Communication, YARC: York Assessment of Reading for Comprehension.

Supplementary Table S6. Developmental outcomes of UNHS.

Study	Location / Yield	Study Details					Outcomes					
		Study Design	Setting	Population	Participants	Assessment	Speech	Language	Literacy	Social Dev	Behavioral Prob	Quality of Life
Kennedy et al. (2006) [1]	UK Wessex Yield: 120/168 (71.4%)	Cohort UNHS PCHL vs No UNHS PCHL vs NH Age of ID</> 9 months CA = mean age 7.9 years Range: 5.4 to 11.7	Screen: INP Dev assessment: OUTP	1993–6 157,000 infants screened (87% coverage of births) 1.5% false positive Two-stage TEOAE-AABR	EG: n = 120 PCHL (n = 63 NH; n = 57 UNHS) CG: n=60 NI	TROG [12], BPVS [13], RBST [22] Children's Comm Checklist [25]	No significant difference by UNHS (or age of ID) for Speech	UNHS: significant increased Rec Lang but not Exp Lang Early age of ID increased Rec Lang & Exp Lang PCHL significant;y lower than NH	-	-	-	-
McCann et al. (2009) [14]	UK Wessex Yield: 120/168 (71.4%)	Cohort UNHS vs. No UNHS Age ID < 9 months 0.0 vs. >9 months CA: Mean age 7.9 Range 5.4 to 11.7 years	Screen: INP Dev Assessment: OUTPt	1993–6 157,000 infants screened (87% coverage of births) 1.5% false positive Two-stage TEOAE-AABR	EG: n = 120 UNHS n = 57 No UNHS n = 61 ID < 9 months (n = 57) ID > 9 months (n = 64) CG: n = 63 NH	WORD [16] basic reading WORD [16] RDGCOMP; VABS [15] Comm, TROG[12] Rec BPVS [13] RBST [22] RBST [22] avg 5 longest sentences	-	UNHS significant better No UNHS ID < 9months significant better than ID > 9 months PCHI significant lower than NH	UNHS: significant better than No UNHS	-	-	-
Worsfold et al. (2010) [27]	UK Wessex Yield: 89/168 (53%)	Cohort UNHS vs. No UNHS AgeID < 9 months vs Age ID > 9 months CA = 7.7 years Range 6-6-10-9 years	Screen: INP Dev Assessment: OUTP	1993–6 157,000 infants screened (87% coverage of births) 1.5% false positive Two-stage TEOAE-AABR	EG: n = 89 UNHS n = 44 No UNHS n = 44 Spoken Lang only Age of ID < 9 months n = 49 Age of ID > 9 months n = 40 CG: n = 63 NH	Lang sample: LARSP [26]	-	UNHS vs No UNHS not significant No difference low pitch morphology markers, phonological simplification, Multiple clauses Age of ID < 9 months significant more sentences, categories high pitched morphological markers OR 3.03 if confirmed early	-	-	-	-

Stevenson et al. (2010) [27]	UK<br>Wessex<br>Yield:<br>120/168<br>(71%)	Cohort:<br>AgeID < 9 months vs Age ID > 8 months<br>CA = Mean 7-7 years<br>Range 5-5 years-11-8 years	Screen: INP<br>Dev Assessment: OUTPt	1993–6<br>157,000 infants screened<br>(87% coverage of births)<br>1.5% false positive<br>Two-stage TEOAE-AABR	EG: UNHS *n* = 57<br>No UNHS *n* = 61<br>Age of ID < 9 months (*n* = 57) vs Age of ID > 9 months (*n* = 64)<br>CG: NH = 63	TROG [12], BPVS [13], RCPM [28], SDQ [29], Teacher/ Parent, VABS [15]	-	Lang ID < 9 months significant better than ID > 9 months<br>Lang PCHL 1.5 SD lower than NH		PCHL significant lower SS than NH on daily living skills and socialization	Age ID < 9 months vs AgeID > 9 months no significant difference behavior problems<br>PCHL: Higher Total Behavior Probs than NH<br>NML Lang: Lower behavior problems	-
Stevenson et al. (2018) [30]	UK<br>Wessex<br>Yield:<br>76/168<br>(45%)	Cohort<br>UNHS vs. No UNHS & Age of ID < 9 months vs Age of ID > 9 months<br>Lang reading at CA = 6–10 years<br>Emotional &Behavior at 13–20 years	Screen: INP<br>Dev Assessment: OUTP	1993–6<br>157,000 infants screened<br>(87% coverage of births)<br>1.5% false positive<br>Two-stage TEOAE-AABR	EG: *n* = 76<br>PCHL,<br>CG NH: *n* = 37	TROG [12], BPVS [13], VABS [15]. WORD [16], YARC [18], RCPM [28], SDQ [29]	-	Lang & reading at 6–10 predicts Emotional and Behavior probs at 13–20 years	UNHS & Age ID < 9 months predict Lang 6–10 years	PCHL significant lower on daily living and socialization than NH	PCHL significant higher total behavior probs than NH<br>NML Lang = NML behavior probs	-
Pimperton et al. (2016) [17]	UK<br>Wessex<br>Yield:<br>76/168<br>(45%)<br>76/120<br>(63% of initial cohort)	Cohort<br>UNHS vs. No UNHS & AgeID < 9 months vs Age ID > 9 months<br>CA:13–19 years	Screen: INP<br>Dev Assessment: OUTP	1993–6<br>157,000 infants screened<br>(87% coverage of births<br>1.5% false positive<br>Two-stage TEOAE-AABR	EG: *n* = 76<br>PCHI<br>UNHS *n* = 37<br>No UNHS *n* = 39<br>AgeID < 9 months *n* = 35<br>AgeID > 9 months *n* = 41<br>Control: *n* = 38 NH	YARC [18]	-	AgeID < 9 months<br>½ SD lower than NH<br>AgeID > 9 months almost 2 SD below<br>AgeID < 9 months, HL, MLE Cog predict reading	UNHS predicts Age ID<br>AgeID < 9 months predicts reading<br>If UNHS& confirmed <9 months-maintained literacy gap from 7 years.<br>AgeID > 9 months increased gap	-	-	-
Wake et al. (2016) [7]	Australia: Victoria &NSW<br>Yield:<br>134/313<br>(43%)	Cohort (Quasi-randomized)<br>UNHS, RFS,<br>No UNHS<br>CA: UNHS & RFS 5 & 6 years<br>No UNHS: 7 & 8 years	Screen: INP<br>Dev Assessments OUTP	2003–2005<br>172 523 babies NSW<br>123 855 Victoria.	EG: UNHS: NSW 2003–5 *n* = 69<br>RFS: Victoria 2003–5 *n* = 65<br>CG: No UNHS: Victoria 1991–3	SDQ [29], PQOL [31], PLS-4 [20], PPVT-4 [21]	-	Rec Lang: PLS-4<br>UNHS>RFS& No UNHS<br>UNHS: 88.9 vs RFS: 83, No UNHS:81.8<br>Exp Lang: PLS-4 [20]<br>UNHS >RFS& No UNHS	-	-	UNHS vs No UNHS not significant Behavior (SDQ)<br>AgeID < 9 months not significant<br>AgeID > 9 months	UNHS vs No UNHS not significant (PQOL)<br>AgeID < 9 months not significant

	179 non partici-pants				n = 86			UNHS: 89.3 vs RFS:80.7 vs No UNHS: 74.9 Rec Voc (PPVT-4) [21] UNHS>RFS & No UNHS UNHS: 91.5, RFS: 83.8, No UNHS: 79.4				AgeID > 9 months
Korver et al. (2010) [32]	Nether-lands 65 regs	Cohorts: UNHS vs DS at 9 months AgeID UNHS is late CA: 3–5 years	Screen: INP Dev Assess-ments OUTP	2002–2006 UNHS: n = 335,560 DS: n = 234,826	EG: UNHS n = 80 DS n = 70 No CG	CDI [19] Mac CDI [33] PQOL [31]	-	UNHS higher (CDI ELQ[19]) = 82.4 DS=76 Not stat significant UNHS: CDI [19] LCQ = 75.4 DS:72.7 Not stat sig-nificant No significant diff MAC-CDI [33]	-	UNHS signif-icant higher than DS (CDI [19] SQ) 8.8 higher UNHS CDI [19] SQ = 79.9	-	PQOL [31]: UNHS 5.3Qs higher than DS UNHS = 85.8 DS = 80.5
Sininger et al. (2010) [34]	USA UNHS predict earlier age of fit	Cohorts Age at Fit DOHL NCAST [35] (parent/infant integration) Language Home Educ CA: 3–5 years	Screen: INP Dev Assess-ments: OUTP	No info on UNHS popula-tion	EG: n = 44 of 64 n = 40 English speaking n = 4 Spanish speaking n = 14 bilingual (Spanish, ASL, Chinese) NoCG	Ped Speech Intel-ligibility [36]; OLIMSPAC [37]; Arizona Articula-tion Test [38] Reynell Dev Lang Scales RDLS [39]	Age of fit pred PSI [36] & OLIMSPAC [37] DOHL & Age fit pred Arizona [38]	HL Implant age predicted RDSL [39] Age of HA fit each month lag attributed to lang lag 0.17 month receptive and 0.30 lag Exp Lang Test	-	-	-	
Yoshinaga-Itano et al. (2000) [4]	USA: Colorado	Cohort: UNHS vs No UNHS UNHS+PRUNHS vs No UNHS vs PR No UNHS Vs prior to UNHS CA 9–61 months	Screen: INP Dev Assess-ment: OUTP	148,240 infants screened 1992–1999 n = 54 45% of all BHL UNHS 1992–1998 Mehl & Thom-son 2002 [6]	EG: UNHS: n = 25 EG: PRUNHS: Parent report n = 29 CG1: No UNHS: n = 25 CG2: PRNS n = 77 CG3: Born before 1992 n = 93	CDI [19] MAC CDI [33] Language sample Speech Intelligi-bility Rating Scale, # conso-nant, # consonant blend, # intelligi-ble words, # diff words	Significant higher CDI [31], MAC-CDI [33] quo-tients, UNHS sig-nificant more consonants & sig-nificant better speech intelligi-bility, more con-sonants, conso-nant blends, intel-ligible words, diff words	Screened children are 2.5 times more likely to have language quotients in the normal range (>80) when compared to no screen group and two times more likely than the pre-UNHS group				
Yoshinaga-Itano et al. (2001) [5]	USA: Colorado	Cohort: UNHS vs No UNHS UNHS+PRUNHS vs No UNHS vs PR No UNHS Vs prior to UNHS CA 9–61 months	Screen: INP Dev Assess-ment: OUTP	148,240 infants screened 1992–1999 n = 54 45% of all BHL UNHS 1992–1998	UNHS: n = 25 PRUNHS: Par-ent report n = 29 CG1: No UNHS: n = 25	MACCDI [33] CDI [19] Language sample	UNHS significant more consonants & significant bet-ter speech intelli-gibility	CDI [19] UNHS: LCQ = 82.9 n. UNHS LCQ = 62.1 UNHS EL: ELQ = 81.5 No UNHS: ELQ = 66.8	-	-	-	-

				Mehl & Thomson 2002 [6]	CG2: PRNS<br>*n* = 77<br>CG3: Born before 1992<br>*n* = 93			MACCDI [33]<br>UNHS + 95.5 word mean<br>No UNHS = 14.5 word mean<br>UNHS #diff words: 30<br>No UNHS #diff words: 7<br>Born before 1992: 55.7 LQ				
Yoshinaga-Itano et al. (2020) [24]	USA National 12 states	Cohorts:<br>EHDI 1–3–6 (screen by 1 months, identify by 3 months and in intervention by 6 months) vs not EHDI 1–3–6 (1–3, 1–6 and 1 only)<br>CA = 8–39 months<br>Mean age: 25.3 months	Screen: INP Dev Assessment OUTP	No info on # infants screened	*n* = 448<br>UNHS: *n* = 258<br>No UNHS *n* = 190	MacCDI [33]	-	EHDI 1-3-6= EVQ = 79.2<br>1–3 = EVQ = 66.7<br>1–6= EVQ = 68.7<br>1 = EVQ = 68.9<br>EHDI 1-3-6 significant higher than not EHDI 1-3-6(1-3,1-6, 1)<br>No significant diffs EHDI 1, EHDI 1–3, or EHDI 1–6	-	-	-	-

AgeID: Age of Identification; AMP: Amplification, ASL: A,merican Sign Language, BHL: Bilateral hearing loss, BPVT: British Picture Vocabulary Test, CA: Chronological age, CDI: Child Development Inventory, CG: Control Group, CQ: Cognitive Quotient, Cog: Cognitive; DOHL: Degree of Hearing Loss; DS: Distraction Screen, EG: Experimental Group; EHDI 1 (Screen by 1 months), EHDI 1–3 (screen by 1 months, identify by 3 months), EHDI 1-3-6 (Screen by 1, Identify by 3, in Rx by 6 months), EHDI 1–6 (screen by 1 months, Rx by 6 months), ELQ: Expressive Language Quotient; EVQ: Expressive Vocabulary Quotient,Exp Lang: Expressive Language; PSI: Parental Stress Inventory, LQ: Language Quotient; HA: Hearing Aid Fit, ID: Identification, HS: Hearing screening; MacCDI: MacArthur-Bates Communicative Development Inventories, MLE: Mother's level of education, NCAST: NH=Normal Hearing; NML: Normal; NS: No Screen, NSW: New South Wales; OLIMSPAC: On-line Imitative Test of Speech Pattern Contrast Perception, RLQ: Receptive Language Quotient; PCHL: Permanent childhood hearing loss, EG: Experimental Group, PCHL: Permanent congenital hearing loss, PLS-4: Preschool Language Scale - Fourth Edition, PPVT-4: Peabody Picture Vocabulary Test-4, PRNUNHS: Parent Report No UNHS, PRUNHS: Parent Report UNHS, RBST: Renfrow Bus Story Test, RDSL: Reynell Development Scales of Language, RFS: Risk Factor Screen, Rec Lang: Receptive Language; Rec Voc: Receptive Vocabulary; RX: Intervention, SDQ: Strengths and Difficulties Questionnaire; TEOAE-AABR: Transient evoked otoacoustic emissions-Automated auditory brain stem response; SD: standard deviation; SQ: Social Quotient; SS: Standard Scores; TROG: Test for Reception of Grammar, UHL: Unilateral hearing loss, UK: United Kingdom; UNHS: Universal newborn hearing screening, USA: United States of America, VABS: Vineland Adaptive Behavior Scale, Wechsler LQ: Language quotient, WORD: Wechsler Objective Reading Dimensions.

Supplementary Table S7. Societal cost-benefit of UNHS.

Study	Location	Study details						Cost analysis outcome
		Study design	Measure	Population	Participants	Screen method	Cost outcome	
Schroeder et al. (2006) [40]	UK Wessex study	Economic benefit Comparisons: PCHL vs NH UNHS vs No UNHS AgeID </>9 months Language by z score	Mean societal cost in preceding year at 7–9 years	Cohort study 1992–1997 157,000 births Wessex study	n = 183 CA = 7–9 years n = 120/168 PCHL n = 63 children with NH	UNHS: TEOAE + AABR	Cost was £14,092.5 for PCHL compared to £4206.8 for NH Diff of £9885.7	UNHS = cost reduction of £2213.2 LANG: Mean societal costs reduced by ££2553 for each unit increase in z score for reclang for PCHL. Age of ID </>9 months: Costs similar
Chorozoglou et al. (2018) [41]	UK Wessex study	Economic benefit Comparisons: PCHL vs. NH UNHS vs No UNHS AgeID </>9 months Degree HL: Moderate vs severe vs profound	Resource use and costs preceding 12-month period estimated mean age 16.9 years	Cohort study 1992–1997 157,000 births Wessex	n = 110/183 CA = 13–20 years n = 73/120 PCHL n = 37/63 NH	UNHS: TEOAE+AABR	Mean costs for PCHL £15914 and NH £5883 pounds Diff: £10031 DOHL: Moderate £5916+ Severe £6605+ Profound £18437+	UNHS not significant lower overall costs decreased £3596 LANG: Increase of one unit rec language z-score decrease £1616 ADD MED COND: increased £15385+
Mehl & Thomson (2002) [6]	USA: Colorado population	Estimated economic benefit Comparison: UNHS vs No UNHS	Cost of educational service	Estimated population: 100,000	Assumed all children with PCHL would be identified early with no loss to follow-up	UNHS: AABR or TEOAE or both	UNHS vs No UNHS: Potential cost savings: 37% reduction ed cost $9308	Expected reductions ed costs would exceed cost of UNHS within 12 years
Keren et al. (2002) [42]	USA	Estimated economic benefit Comparison: UNHS vs. No UNHS vs selective screen	Societal costs including health, education, lifetime productivity	Estimated population: 80,000	Assumed 50–70% children receive RX by 12 months would have language in typical range, compared with 28–40% of id later. Assumed language typical range = 10% reduction in excess cost ed and 7% reduction lost productivity	Various technologies	Detection/deaf infant: $69,200 No UNHS, $671,200 SS,$ 2,122,700 UNHS. Age of ID by 6 months: deaf: $2,300 NS, $10,100 SS and $21,400 UNHS. Cost NML LANG: $2,215,500 No UNHS, $1,978,100 SS $1,769,300 for UNHS.	Children who have “normal” language have 10% lower excess cost of education and 75% lower productivity losses

AABR: automated auditory brainstem response, ADD MED COND: Additional medical condition, AgeID: Age of identification, CA: Chronological Age, LANG: Language, NH: Normal hearing, No UNHS: no Universal newborn hearing screening, PCHL: Permanent Congenital Hearing Loss, RECLANG: Receptive language, TEOAE: transient evoked otoacoustic emissions, UK: United Kingdom; UNHS: Universal newborn hearing screening, USA: United States of America.

Supplementary Table S8. Negative side effects (harm) of UNHS.

Study	Location	Study Details							Outcome
		Study Design	Setting	Population	Participants	Assessment Timing	Measure	Yield	
Kennedy (1998) [43]	UK	Cohort Comparison: positive vs negative screen	Screen: hospital Dev Ass: outpatient	1992–97 157,000 births Wessex TEOAE + AABR	*n* = 100 positive screen *n* = 100 negative screen	2 to 12 months after screen Questionnaires sent over 18 month period	Spielberger State-Trait Anxiety Inventory (STAI) [44] and the Attitude towards the Baby Scale	Completed returns received from 50–60% of the sample. telephone contact increased %age to 75% of each group	No significant diff screen neg vs screen pos Anxiety trait and state scores very similar to population sample of women of child-bearing age.
Weichbold & Welzl Mueller (2001) [8]	Austria	Cohort Comparison: Fail 1st screen, Fail 2nd screen	Screen: hospital Dev Ass: outpatient	1999–2000 *n* = 6000 newborns screened Two-stage TEOAE	*n* = 85 fail 1st screen *n* = 51 knew 1st screen fail *n*=34 2nd screen fail	Immediately after receiving fail screen result 1st & 2nd screen	First screen: -Were you present? -Did you learn need for retest -Were you worried? 2nd screen: -How much have you been worried? -Did you feel sufficiently informed that infant not very likely to be hearing impaired	80% of screened 70% follow-up rate 85/95 false positive consented 51/95 (60%) knew screen result	1st screen: 59% (failed screen) were not concerned 27% slightly concerned 14% highly concerned. 2nd test: 42% not concerned 37% slightly concerned, 21% highly concerned. No significant difference between groups
Tueller & White (2016) [45] /Tueller (2006) [46]	USA: Utah	Cohort Comparison: Time 1: 1st screen vs. Time 2 2nd screen	Screen: hospital Assessments: questionnaire mailed	Infants screened: 2004: 31,410 2005: 7,231 TEOAE	Total: *n* = 286 Time1 1st screen: *n* = 192 *n* = 83 pass *n* = 34 fail/pass *n* = 9 Fail/Fail *n* = 66 result unknown Time 2: *n* = 95 *n* = 60 pass *n* = 18 Fail/pass *n* = 7 FailFail *n* = 10 result unknown	Time 1: 1 week after discharge Time 2: 6 weeks	Infant Health Concerns Scale State Trait Anxiety Inventory [44] Child Vulnerability Scale scores	*n* = 286 invited Time 1: *n* = 192 (67% return rate) Time 2: *n* = 95 (49% of initial)	Mothers worried more about many other aspects of infant health/behavior than about hearing. False positive-initially more worried than other aspects of infant's health, disappeared in 6 weeks. No significant diffs Time 1 and Time 2 STAI [44] Pass vs Fail/refer
Watkin et al. (1998) [47]	UK Whipps Cross Hospital Maternity Unit	Cohort Fail in both ears, Fail in one ear, Pass Initial questionnaires was given to a sample	Screen: hospital Assessments: Questionnaires mailed	Infants screened: 4500/ years 290/4500 TEOAE	*n* = 290 *n* = 288/290 enrolled initial TEOAE Two declined *n* = 49 failed both ears *n* = 79 failed one ear	Stage 1: 0–3 days Stage 2: 6 weeks Stage 3: 9 months Control vs. fails	Sociodemography Deaf Awareness Screen Satisfaction, Screen Attitudes, Anxiety Assessment STAI [44]	Stage 1: *n* = 288/290 (99% return rate) infants 0–3 days Stage 2 *n* = 57 (60% return rate) mothers, infants 6 weeks old Control: *n* = 61/102 (61% return rate) mothers Stage 3—infants 9 months	No significant difference retest vs control: STAI [44] No significant difference at 9 months STAI [44] from initial *n* = 288 at birth. 1% worried

Crockett et al. (2006) [48]	UK	Cohort<br>Random sample<br>Grp 1: 1st & 2nd Pass<br>Grp 2: Pass Screen 3,<br>Grp 3: Fail/refer 1 ear,<br>Grp 4: Fail/refer both ears	Screen: Hospital Questionnaire mailed	No info on # infants screened TEOAE	Grp 1: $n = 103$<br>Pass 1st & 2nd screen<br>Grp 2: $n = 81$<br>Pass 3rd screen<br>Grp 3: $n = 105$<br>Fail/refer aud dx one ear 3rd screen<br>Grp 4: $n = 55$<br>Fail/refer aud dx both ears	Timing: 3 weeks after completing screen	Questionnaires<br>STAI[44]<br>Worry about baby's hearing<br>Certainty about baby's hearing<br>Knowledge about NHSP	53% response rate<br>$n = 344/722$	Normal Range: Mean anxiety/worry<br>Significant trend increase anxiety/worry w/ # test<br>significant interaction between mothers' group & understanding that receipt of refer/fail unlikely to mean that baby had a hearing loss<br>Mothers in group 4 w/ this understanding had lower anxiety
Crockett et al. (2005) [49]	UK	Cohort<br>Health visitors randomly selected to select cohort<br>Comparison: UNHS vs HVDT	Screen: hospital Questionnaires: mailed	No info on # infants screened TEOAE + AABR HVDT	Invited: First 35 w/pass<br>First 30 w/refer<br>$n = 27$ pass<br>n=21 recalled after HVDT	Timing: 3 weeks after screen	Short form Spielberger State-Trait Anxiety Inventory [44] (Maternal state anxiety)<br>Worry about baby's hearing<br>Certainty about baby's hearing<br>Satisfaction with screening test<br>Attitude to screening test	67% (67/99) (HVDT) returned first questionnaire<br>48% (HVDT) returned at both time periods (49% pass, 48% refer)<br>35% of total sample: HVDT + UNHS	No significant diffs in maternal anxiety/worry & certainty<br>UNHS: Higher satisfaction than HVDT. UNHS: Higher positive attitudes after satisfactory screen result than HVDT
Vohr et al. (2001) [50]	USA: Rhode Island	Cohort<br>Population<br>6/1-10/15 1997<br>7/1 = 8/30 1999<br>Comparison: 1st screen vs 2nd screen	Screen: hospital Questionnaires: hospital	Infants screened: $n = 384 + 44 = 428$ TEOAE + AABR	$n = 307$ mothers at time of first screen<br>$n = 40$ mothers at 2nd screen	Timing: immediately after screen	Questionnaires about social, environmental, family factors, knowledge about the hearing screen program, worry about hearing screening program	80% of mothers with first screen and 90% of rescreen agreed to participate	No or mild worry: 88–89%<br>Somewhat worried: 7–8%<br>Worried: 1.3–2.5%<br>Very worried: 1.3–2.7% .<br>Greater worry 2nd screen<br>Socio-econ disadvantage: greater risk of increased worry and less aware of NHS.
Kolski et al. (2007) [51]	France	Cohort<br>Comparison: UNHS vs 2 months screen vs No UNHS<br>UNHS 1st screen vs 2nd screen	Screen: hospital Interviews: hospital 1st INP, 2nd OUTP	5,790 (3202 1st strategy, 2588 2nd strategy)<br>2nd strategy: 1661/2588 (61%) screened<br>OAE screen initial and retest	$n = 115$ infants screened,<br>UNHS: $n = 58$<br>Screen at 2 months: $n = 57$<br>No screen: $n = 28$	Two semi-directive interviews<br>1st interview after results provided to mother<br>2nd interview<br>After 1st screen before confirmation test several weeks later	MADRS [52]scale: dimensions of post-partum depression<br>Anxiety scale from EPDS questionnaire (Edinburgh post-natal depression scale [53]) and certain items of Kennerley self-administered questionnaire<br>Interaction scale—investigates visual, physical, mental and social dimensions of mother-infant relationships	143 of 5790 screened (2.5%)<br>Coverage:<br>95% screened UNHS<br>61% screened 2 months	UNHS vs 2 months.: No significant diff MADRS [44], quality early interactions<br>Fail/refer: increased anxiety, decreased psychological state which affected quality of early interactions<br>Fail/refer vs. No UNHS: No significant diff MADRS [52] depression, anxiety<br>Fail/refer vs. No UNHS vs 2 months:<br>No significant diff fail/refer vs No UNHS regardless of time of screening

Khairi et al. (2011) [54]	Malaysia Hospital Universiti Sains Malaysia (HUSM)	Cohort Two stage OAE Retest OAE 6 weeks later at follow-up clinics	Screen: hospital 1st interview: hospital 2nd interview ORL clinic	No info on # infants screened OAE screen	n = 78 fail/refer first screening n = 50 2nd screening	Timing: 1st face-to-face interview immediately after mother informed about referral result 1st screen 2nd face-to-face before second screen 6 weeks after 1st	Malay translation Beck Anxiety Inventory [55]	No info about # contacted n = 78 (consented 1st screen) n=50 (64% returned 2nd screen)	74% had mild anxiety that decreased to 68% before 1st and 2nd screening. Moderate anxiety—10% 8% had severe anxiety (18%)—1st screen—reduced to 14% moderate-severe, (4% severe) before 2nd screen
Stuart, Moretz & Yang (2000) [56]	USA Pitt County Memorial Hospital, Greenville, NC WBN	Random selection Comparison: MWIP (mothers w/infants who pass) vs MWIF (mothers w/infants who fail)	Screen: hospital Questionnaire: Telephone interview	No info on # infants screened AABR screen	n = 20 (MWIP) vs. n = 20 (MWIF)	Timing: 1–2 weeks after discharge Telephone interview	PSI: Parenting Stress Index [57]	No info # screened and % invited, % enrolled	No significant diff Total stress, life stress, child domain, parent domain. MWIP lower than MWIF MWIP and MWIF experience equivalent stress levels

AABR: Automated auditory brain stem response, EPDS: Edinburg Post Natal Depression Scale, HUSM: Hospital Universiti Sains Malaysia, HVDT: Health Visitor Distraction Test, MADRS: Montgomery-Asberg Depression Rating Scale, MWIF: Mothers with infants who failed, MWIP: Mothers with infants who passed, NC: North Carolina, No UNHS: No universal newborn hearing screening, PSI: Parenting Stress Index, STAI: Spielberger State Trait Anxiety Inventory, TEOAE: Transient evoked otoacoustic emissions, UK: United Kingdom, UNHS: Universal newborn hearing screening, USA: United States of America, WBN: Well Baby Nursery.

1. Kennedy, C.R.; McCann, D.C.; Campbell, M.J.; Law, C.M.; Mullee, M.; Petrou, S.; Watkin, P.; Worsfold, S.; Yuen, H.M.; Stevenson, J. Language ability after early detection of permanent childhood hearing impairment. *N. Engl. J. Med.* **2006**, *354*, 2131–2141, doi:10.1056/nejmoa054915.
2. Uus, K.; Bamford, J. Effectiveness of population-based newborn hearing screening in England: Ages of interventions and profile of cases. *Pediatrics* **2006**, *117*, e887–e893, doi:10.1542/peds.2005-1064.
3. Wood, S.A.; Sutton, G.J.; Davis, A.C. Performance and characteristics of the newborn hearing screening programme in England: The first seven years. *Int. J. Audiol.* **2015**, *54*, 353–358, doi:10.3109/14992027.2014.989548.
4. Yoshinaga-Itano, C.; Coulter, D.; Thomson, V. The Colorado Newborn Hearing Screening Project: Effects on speech and language development for children with hearing loss. *J. Perinatol.* **2000**, *20*, S132–S137, doi:10.1038/sj.jp.7200438.
5. Yoshinaga-Itano, C.; Coulter, D.; Thomson, V. Developmental outcomes of children with hearing loss born in Colorado hospitals with and without universal newborn hearing screening programs. *Semin. Neonatol.* **2001**, *6*, 521–529, doi:10.1053/siny.2001.0075.
6. Mehl, A.L.; Thomson, V. The Colorado Newborn Hearing Screening Project, 1992-1999: On the threshold of effective population-based universal newborn hearing screening. *Pediatrics* **2002**, *109*, e7, doi:10.1542/peds.109.1.e7.
7. Wake, M.; Ching, T.Y.C.; Wirth, K.; Poulakis, Z.; Mensah, F.K.; Gold, L.; King, A.; Bryson, H.E.; Reilly, S.; Rickards, F. Population outcomes of three approaches to detection of congenital hearing loss. *Pediatrics* **2016**, *137*, e20151722, doi:10.1542/peds.2015-1722.
8. Weichbold, V.; Welzl-Mueller, K. Maternal concern about positive test results in universal newborn hearing screening. *Pediatrics* **2001**, *108*, 1111–1116, doi:10.1542/peds.108.5.1111.
9. Sininger, Y.S.; Martinez, A.; Eisenberg, L.; Christensen, E.; Grimes, A.; Hu, J. Newborn hearing screening speeds diagnosis and access to intervention by 20–25 months. *J. Am. Acad. Audiol.* **2009**, *20*, 049–057, doi:10.3766/jaaa.20.1.5.

10. Dalzell, L.; Orlando, M.; MacDonald, M.; Berg, A.; Bradley, M.; Cacace, A.; Campbell, D.; DeCristofaro, J.; Gravel, J.; Greenberg, E.; et al. The New York State universal newborn hearing screening demonstration project: Ages of hearing loss identification, hearing aid fitting, and enrollment in early intervention. *Ear Hear.* **2000**, *21*, 118–130, doi:10.1097/00003446-200004000-00006.
11. Yoshinaga-Itano, C.; Sedey, A.L.; Coulter, D.K.; Mehl, A.L. Language of Early- and Later-identified Children With Hearing Loss. *Pediatr.* **1998**, *102*, 1161–1171, doi:10.1542/peds.102.5.1161.
12. Bishop, D.V.M. *Test for reception of grammar*. Manchester, United Kingdom: Age and Cognitive Performance Research Centre, University of Manchester, 1983.
13. Dunn, L.M.; Whetton, C.; Burley, J. *British Picture Vocabulary Scale,* 2nd ed.; NFER-Nelson: Windsor, United Kingdom, 1997.
14. McCann, D.C.; Worsfold, S.; Law, C.M.; Mullee, M.; Petrou, S.; Stevenson, J.; Yuen, H.M.; Kennedy, C.R. Reading and communication skills after universal newborn screening for permanent childhood hearing impairment. *Arch. Dis. Child.* **2008**, *94*, 293–297, doi:10.1136/adc.2008.151217.
15. Sparrow, S.S.; Balla, D.A.; Cicchetti, D.V. *Vineland: Adaptive Behavior: Scales, Interview edition: Survey form manual*. American Guidance Service: Circle Pines, MN, USA, 1984.
16. Wechsler, D. *Wechsler Objective Reading Dimensions*. The Psychological Corporation: London, United Kingdom, 2003.
17. Pimperton, H.; Blythe, H.; Kreppner, J.; Mahon, M.; Peacock, J.L.; Stevenson, J.; Terlektsi, E.; Worsfold, S.; Yuen, H.M.; Kennedy, C.R. The impact of universal newborn hearing screening on long-term literacy outcomes: A prospective cohort study. *Arch. Dis. Child.* **2016**, *101*, 9–15, doi:10.1136/archdischild-2014-307516.
18. Stothard, S.E.; Hulme, C.; Clarke, P.; Bowyer-Crane; C., Harrington; A., Truelove, E.; Nation, K. *YARC York Assessment of Reading for Comprehension Secondary*. GL Assessment: London, United Kingdom, 2010.
19. Ireton, H.; Thwing, E. The Minnesota Child Development Inventory in the psychiatric-developmental evaluation of the preschool-age child. *Child. Psychiatry Hum. Dev.* **1972**, *3*, 102–114, doi:10.1007/bf01433451.
20. Zimmerman, I.L.; Steiner, V.G., Pond, R.E. *Preschool Language Scale*, 4th ed.; Harcourt Assessment: London, United Kingdom, 2002.
21. Dunn, L.M.; Dunn, D.M. *The Peabody Picture Vocabulary Test*, 4th ed.; Pearson: Minneapolis, MN, USA, 2007.
22. Renfrew, C. *Renfrew Bus Story Manual: A test of narrative speech*, 3rd ed.; Winslow Press: Oxford, United Kingdom, 1995.
23. Worsfold, S.; Mahon, M.; Yuen, H.M.; Kennedy, C. Narrative skills following early confirmation of permanent childhood hearing impairment. *Dev. Med. Child. Neurol.* **2010**, *52*, 922–928, doi:10.1111/j.1469-8749.2010.03641.x.
24. Yoshinaga-Itano, C.; Hunnicutt, C.; Manchaiah, V. A Systematic Review of the Evidence for the Effectiveness of Universal Newborn Hearing Screening. *PROSPERO* **2020**, CRD42020175451.
25. Bishop, D.V. Development of the Children's Communication Checklist (CCC): A method for assessing qualitative aspects of communicative impairment in children. *J. Child. Psychol. Psychiatry* **1998**, *39*, 879–891.
26. Crystal, D.; Fletcher, P.; Garman, M. *The grammatical analysis of language disability—a procedure for assessment and remediation*, 1st ed.; Edward Arnold: London, United Kingdom, 1976.
27. Stevenson, J.; McCann, D.C.; Law, C.M.; Mullee, M.; Petrou, S.; Worsfold, S.; Yuen, H.M.; Kennedy, C.R. The effect of early confirmation of hearing loss on the behaviour in middle childhood of children with bilateral hearing impairment. *Dev. Med. Child. Neurol.* **2010**, *53*, 269–274, doi:10.1111/j.1469-8749.2010.03839.x.
28. Raven, C.; Raven, J.C.; Court, J.H. *Manual for Raven's Progressive Matrices and Vocabulary Scales*. Oxford Psychologists Press, Oxford, United Kingdom, 1998.
29. Goodman, R. The Strengths and Difficulties Questionnaire: A Research Note. *J. Child. Psychol. Psychiatry* **1997**, *38*, 581–586, doi:10.1111/j.1469-7610.1997.tb01545.x.
30. Stevenson, J.; Pimperton, H.; Kreppner, J.; Worsfold, S.; Terlektsi, E.; Mahon, M.; Kennedy, C. Language and reading comprehension in middle childhood predicts emotional and behaviour difficulties in adolescence for those with permanent childhood hearing loss. *J. Child. Psychol. Psychiatry* **2018**, *59*, 180–190, doi:10.1111/jcpp.12803.
31. Varni, J.W.; Burwinkle, T.M.; Seid, M., Skarr; D. The PedsQL 4.0 as a pediatric population health measure: Feasibility, reliability, and validity. *Ambul. Pediatr.* 2003, 3, 329–341, doi:10.1367/1539-4409(2003)003<0329:tpaapp>2.0.co;2
32. Korver, A.M.H.; Konings, S.; Dekker, F.W.; Beers, M.; Wever, C.C.; Frijns, J.H.M.; Oudesluys-Murphy, A.M.; DECIBEL Collaborative Study Group. Newborn hearing screening vs later hearing screening and developmental outcomes in children with permanent childhood hearing impairment. *JAMA* **2010**, *304*, 1701, doi:10.1001/jama.2010.1501.
33. Fenson, L.; Marchman, V.A.; Thal, D.; Dale, P.S.; Bates, E., Reznick, J.S. *The MacArthur-Bates Communicative Development Inventories: User's Guide and Technical Manual*, 2nd ed.; Paul, H. Brookes: Baltimore, MD, USA, 2007.
34. Sininger, Y.S.; Grimes, A.; Christensen, E. Auditory development in early amplified children: Factors influencing auditory-based communication outcomes in children with hearing loss. *Ear Hear.* **2010**, *31*, 166–185, doi:10.1097/aud.0b013e3181c8e7b6.
35. Sumner, G.; Spietz, A. *NCAST Caregiver/Parent-Child Interaction Teaching Manual*. NCAST/Caregiver: Seattle, WA, USA, 1994.

36. Jerger, S.; Lewis, S.; Hawkins, J.; Jerger, J. Pediatric speech intelligibility test. I. Generation of test materials. *Int. J. Pediatr. Otorhinolaryngol.* **1980**, *2*, 217–230, doi:10.1016/0165-5876(80)90047-6.
37. Boothroyd, A.; Eisenberg, L.S.; Martinez, A.S. *OLIMSPAC Version 3.1d.* House Ear Institute: Los Angeles, CA, USA, 2005.
38. Fudala, J. *Arizona Test of Articulation-3*. PRO-Ed, Inc: Austin, TX, USA, 2000.
39. Edwards, S.; Fletcher, P.; Garman, M.; Hughes, A.; Letts, C.; Sinda, I. *Reynell Developmental Language Scales* NFER-Nelson Publishing: Windsor, United Kingdom, 1997.
40. Schroeder, L.; Petrou, S.; Kennedy, C.; McCann, D.; Law, C.; Watkin, P.M.; Worsfold, S.; Yuen, H.M. The Economic Costs of Congenital Bilateral Permanent Childhood Hearing Impairment. *Pediatrics* **2006**, *117*, 1101–1112, doi:10.1542/peds.2005-1335.
41. Chorozoglou, M.; Mahon, M.; Pimperton, H.; Worsfold, S.; Kennedy, C.R. Societal costs of permanent childhood hearing loss at teen age: A cross-sectional cohort follow-up study of universal newborn hearing screening. *BMJ Paediatr. Open* **2018**, *2*, e000228, doi:10.1136/bmjpo-2017-000228.
42. Keren, R.; Helfand, M.; Homer, C.; McPhillips, H.; Lieu, T.A. Projected cost-effectiveness of statewide universal newborn hearing screening. P*ediatrics* **2002**, *110*, 855–864, doi:10.1542/peds.110.5.855.
43. Kennedy, C.R.; Kimm, L.; Cafarelli Dees, D.; Campbell, M.J.; Thornton, A.R.D.; Bamber, J.; Innes, V.; Lloyd-Hughes, S.; Parish, R.; Woodhead, C.; et al. Controlled trial of universal neonatal screening for early identification of permanent childhood hearing impairment. *Lancet* **1998**, *352*, 1957–1964, doi:10.1016/s0140-6736(98)06359-4.
44. Marteau, T.M.; Bekker, H. The development of a 6-item short-form of the state scale of the Spielberger State Trait Anxiety In-ventory (STAI). *Br. J. Clin. Psychol.* **1992**; 31, 301–306, doi:10.1111/j.2044-8260.1992.tb00997.x
45. Tueller, S.J.; White, K.R. Maternal anxiety associated with newborn hearing screening. *J. Early Hear. Detect. Interv.* **2016**, *1*, 87–92.
46. Tueller, S.J. Maternal Worry about Infant Health, Maternal Anxiety, and Maternal Perceptions of Child Vulnerability Associated with Newborn Hearing Screening Results. Unpublished master's thesis, Utah State University: Logan, UT, USA, 2006.
47. Watkin, P.M.; Baldwin, M.; Dixon, R.; Beckman, A. Maternal anxiety and attitudes to universal neonatal hearing screening. *Br. J. Audiol.* **1998**, *32*, 27–37, doi:10.3109/03005364000000048.
48. Crockett, R.; Wright, A.J.; Uus, K.; Bamford, J.; Marteau, T.M. Maternal anxiety following newborn hearing screening: The moderating role of knowledge. *J. Med. Screen.* **2006**, *13*, 20–25, doi:10.1258/096914106776179854.
49. Crockett, R.; Baker, H.; Uus, K.; Bamford, J.; Marteau, T.M. Maternal anxiety and satisfaction following infant hearing screening: A comparison of the health visitor distraction test and newborn hearing screening. *J. Med. Screen.* **2005**, *12*, 78–82, doi:10.1258/0969141053908320.
50. Vohr, B.R.; Letourneau, K.S.; McDermott, C. Maternal worry about neonatal hearing screening. *J. Perinatol.* **2001**, *21*, 15–20, doi:10.1038/sj.jp.7200475.
51. Kolski, C.; Le Driant, B.; Lorenzo, P.; Vandromme, L.; Strunski, V. Early hearing screening: What is the best strategy? *Int. J. Pediatr. Otorhinolaryngol* **2007**, *71*, 1055–1060, doi:10.1016/j.ijporl.2007.03.015.
52. Montgomery, S.A.; Asberg, M. A new depression scale designed to be sensitive to change, *Br. J. Psychiat*. **1979**, 134, 382—389, doi:10.1192/bjp.134.4.382.
53. Cox, J.L.; Holden, J.M.; Sagovsky, R. Detection of postnatal depression: Development of the 10 items Edinburgh Postnatal Depression Scale, *Br. J. Psychiat*. **1987**; 150, 782–786, doi:10.1192/bjp.150.6.782.
54. Khairi, M.D.M.; Rafidah, K.N.; Affizal, A.; Normastura, A.R.; Suzana, M.; Normani, Z.M. Anxiety of the Mothers with Referred Baby during Universal Newborn Hearing Screening. *Int. J. Pediatric Otorhinolaryngol.* **2011**, *75*, 513–517, doi:10.1016/j.ijporl.2011.01.009.
55. Beck, A.T.; Epstein, N.; Brown, G.; Steer, R.A. An inventory for measuring clinical anxiety: Psychometric properties, *J. Consult. Clin. Psychol.* **1988,** 56, 893-897, doi:10.1037//0022-006x.56.6.893
56. Stuart, A.; Moretz, M.; Yang, E.Y. An investigation of maternal stress after neonatal hearing screening. *Am. J. Audiol.* **2000**, *9*, 135–141, doi:10.1044/1059-0889(2000/016).
57. Abidin, R.R. *The Parenting Stress Index: Manual*, *3rd ed*. Psychological Assessment Resources: Odessa, FL, USA, 1995.
